# *Taiwanoshaira* Lee & Beenen, a new genus and first record of moss-inhabiting Galerucinae*sensu stricto* (Coleoptera, Chrysomelidae) from Taiwan

**DOI:** 10.3897/zookeys.944.53099

**Published:** 2020-06-30

**Authors:** Chi-Feng Lee, Ron Beenen

**Affiliations:** 1 Applied Zoology Division, Taiwan Agricultural Research Institute, Taichung 413, Taiwan Taiwan Agricultural Research Institute Taichung Taiwan; 2 Martinus Nijhoffhove 51, NL-3437 ZP Nieuwegein, The Netherlands Unaffiliated Leiden Netherlands

**Keywords:** cloud forest, leaf beetle, Malaise trap, moss, nocturnal, taxonomy

## Abstract

*Taiwanoshaira* Lee & Beenen **gen. nov.** is described. It represents the first genus of Galerucinae (s. str.) in Taiwan documented to inhabit moss cushions. *Shaira chujoi* Kimoto, 1982 is transferred to *Taiwanoshaira*, as follows: *T. chujoi* (Kimoto), **comb. nov**. Two new species, *T. taipingshanensis***sp. nov.** and *T. tsoui***sp. nov.**, are described. Adults of *T. taipingshanensis***sp. nov.** were observed feeding on the moss species *Plagiomnium vesicatum* (Besch.) T.J. Kop. (Mniaceae). Microhabitats and distribution of *Taiwanoshaira* species are discussed.

## Introduction

Moss cushions constitute a special environment inhabited by a limited diversity of leaf beetles. Members of more than 50,000 known species of leaf beetles live mainly on the leaf surface of various flowering plants on which they feed. Konstantinov et al. (2013) reported that 27 leaf beetle species from 14 genera live within moss cushions. All known moss-inhabiting leaf beetles belong to the tribe Alticini, known as flea beetles. Eighteen species and four genera were added to the diversity of moss-inhabiting flea beetles by Konstantinov et al. (2019). But only eight species from four genera were documented actually feeding on mosses (Konstantinov et al. 2019).

**Figure 1.**
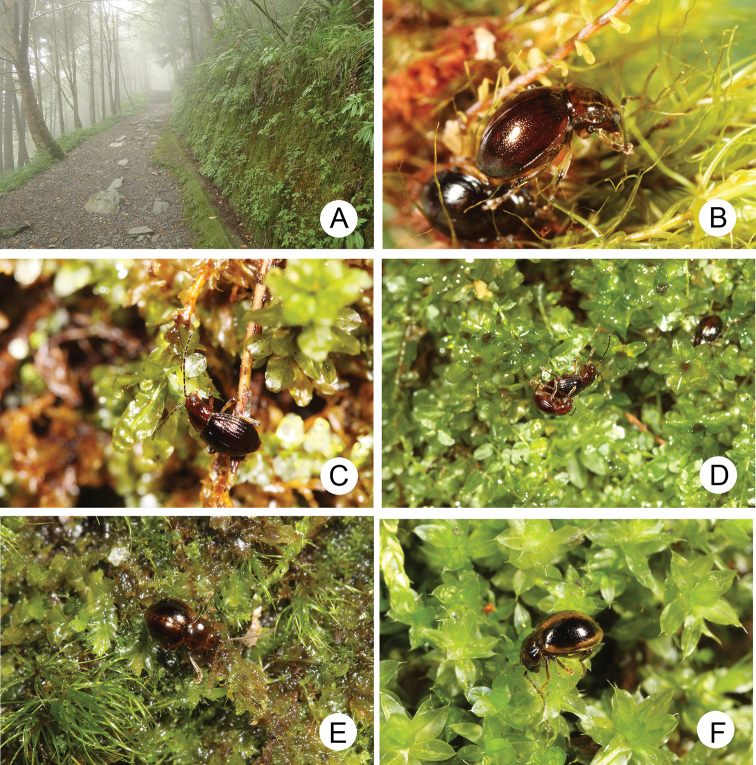
Habitat photographs. **A** microhabitat for *Taiwanoshaira taipingshanensis* sp. nov. and *T. tsoui* sp. nov. at Yuanyang Lake (鴛鴦湖) **B** active adults of *T. chujoi* comb. nov. inside moss cushions at Pilu (畢祿) **C** adult of *T. taipingshanensis* sp. nov. feeding on leaves of *Plagiomnium vesicatum* at Yuanyang Lake (鴛鴦湖) **D** adults of *T. taipingshanensis* sp. nov. mating at Yuanyang Lake (鴛鴦湖) **E** adult of *T. tsoui* sp. nov. at Hsiaofengkou (小風口) **F** adult of *T. tsoui* sp. nov. at Meifeng (梅峰).

The Taiwan Chrysomelid Research Team (TCRT) started their inventory of all species of Chrysomelidae during 2005. We found only adults of *Ivalia* Jacoby inhabited moss cushions early in the project. A TCRT colleague, Sin-Syue Li, found several galerucines (*sensu stricto*) inhabiting moss cushions at Yuanyang Lake (= Yuanyanghu, 鴛鴦湖) (Fig. 1A) on August 19, 2010. During the following year, the first author and several members of TCRT went to the same locality to confirm Li’s observation. We found that adults were nocturnal and active on moss cushions, and observed feeding (Fig. 1C) and mating (Fig. 1D). Catching them by hand-collecting was easy, and more than 30 adults were collected at that time. Collecting proved difficult at other localities even though their behavior was known. They were eventually found on host mosses at only three additional localities, Taipingshan (太平山), Tahsuehshan (大雪山), and Peitungyanshan (北東眼山).

These same moss-inhabiting galerucines were collected using Malaise traps by colleagues at the National Museum of Natural Science at Meifeng (梅峰), Yuanfeng (鳶峰), Hsiaofengkou (小風口), and Bilu Divine Tree (碧綠神木). Malaise traps are mostly used to collect flying insects and the base of the canvas (tent cloth) does normally not touch the ground. When the canvas touches the ground, then beetles that lack flying capacities can crawl upward. Adults of Taiwanese *Paraplotes* species are good examples of flightless insects collected with Malaise traps (Lee 2015). During visits to these localities, more than 150 specimens were captured with hand-collecting at the night.

These galerucines were initially identified as *Shaira chujoi* Kimoto, 1982 and allied undescribed species. However, they were clearly different in diagnostic characters from the type species of the genus, *S. maculata* Maulik, 1936. Thus, generic placement of these species was re-evaluated, species diversity was analyzed, and the results are presented here.

## Material and methods

The abdomens of adults were separated from the bodies and boiled in 10% KOH solution, followed by washing in distilled water to clear and soften genitalia. The genitalia were then dissected from the abdomen, mounted on slides in glycerin, and studied and drawn using a Leica M165 stereomicroscope. For detailed examination a Nikon ECLIPSE 50i microscope was used.

At least two pairs from each species were examined to delimit variability of diagnostic characters. For species collected from more than one locality, at least one pair from each locality was examined. Length was measured from the anterior margin of the eye to the elytral apex, and width at the greatest width of the elytra.

Specimens were available for study and deposited in the following institutions:

**KMNH**Kitakyushu Museum of Natural History and Human History, Kitakyushu, Japan [Yûsuke Minoshima];

**KUEC**Faculty of Agriculture, Kyushu University, Fukuoka, Japan [Osamu Tadauchi];

**NMNS** National Museum of Natural Science, Taichung, Taiwan [Jing-Fu Tsai];

**RBCN** Ron Beenen collection, Nieuwegein, The Netherlands;

**TARI** Applied Zoology Division, Taiwan Agricultural Research Institute, Taichung, Taiwan [Chi-Feng Lee].

Exact label data are cited for all type specimens of previously described species; a double slash (//) divides the data on different labels and a single slash (/) divides the data in different rows. Other comments and remarks are in square brackets: [p] – preceding data are printed, [h] – preceding data are handwritten, [w] – white label, [y] – yellow label, [r] – red label, [b] – blue label.

## Taxonomy

### Chrysomelidae

#### Galerucinae s. str.

##### Taiwanoshaira

Taxon classificationAnimaliaColeopteraChrysomelidae

Lee & Beenen gen. nov.

B1DA222E-DD86-5F83-B8F2-15BF7D857E18

http://zoobank.org/E2A13741-632C-4DB4-B304-C90BC5318FC8

###### Type species.

*Taiwanoshaira tsoui* Lee & Beenen, sp. nov.

###### Description.

Coloration (Figs 3, 6): dark brown or blackish-brown; margins of pronotum and elytra, including suture, yellowish-brown in *T. tsoui* sp. nov.; legs yellowish-brown, but apices of femora and bases of tibiae blackish-brown in *T. chujoi* comb.nov. and *T. tsoui* sp. nov. Body length 4.0–5.7 mm.

Head. Labrum trapezoidal, transverse, with about ten pairs of pores in a transverse row bearing pale, short or long setae, anterior margin medially depressed. Anterior part of head short, almost impunctate and glabrous, lined with setae along anterofrontal ridge. Compound eyes small, interantennal space 3.1–3.6× as wide as diameter of antennal insertion. Frontal tubercles transverse, subtriangular, slightly elevated, glabrous. Vertex smooth and glabrous. Antennae filiform, covered with dense setae, antennomere II subequal or a slightly shorter than III; similar in both sexes.

Pronotum 1.61–1.68 times as broad as long, lateral margins slightly rounded, basally narrowed. Disc smooth, with dense, fine punctures bearing tiny setae in *T. taipingshanensis* sp. nov. and *T. chujoi* comb. nov.; setae reduced in *T. tsoui* sp. nov. Anterior, lateral and posterior margins with marginal bead, without setae along margin. Anterior and posterior angles moderately swollen, rectangular; all angles with setigerous pores bearing long pale setae. Two pairs of longitudinal furrows starting from base, one pair deeper and shorter near middle, the other pair longer but shallow near sides. Scutellum subtriangular, impunctate, glabrous, with rounded apex.

**Figure 2.**
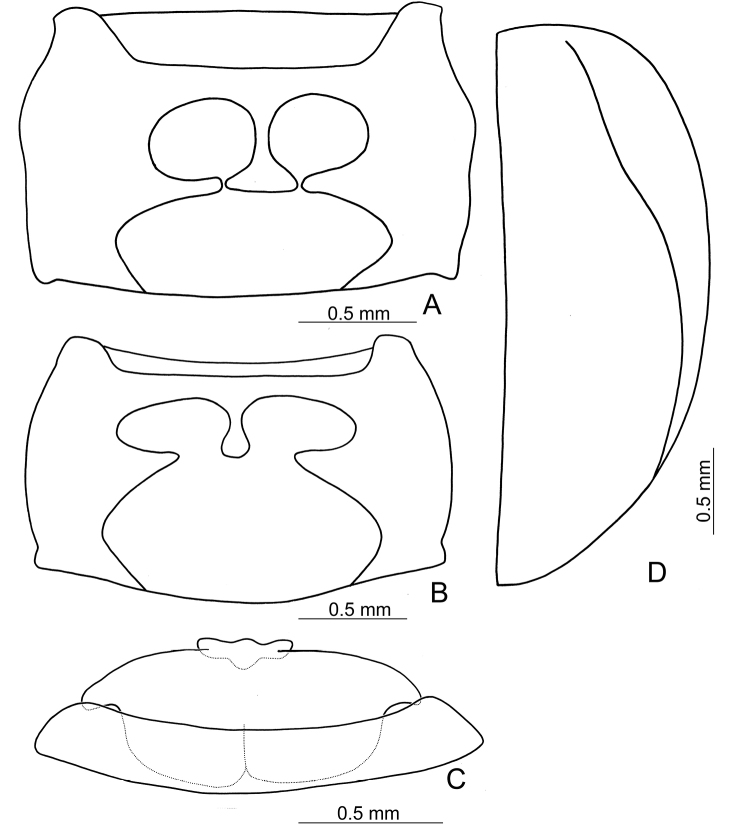
Generic characters for *Taiwanoshaira*. **A** prothorax, ventral view, *T. taipingshanensis* sp. nov. **B** prothorax, ventral view, *T. tsoui* sp. nov. **C** last two abdominal ventrites, dorsal view, male, *T. tsoui* sp. nov. **D** elytron, ventral view, *T. tsoui* sp. nov.

**Figure 3.**
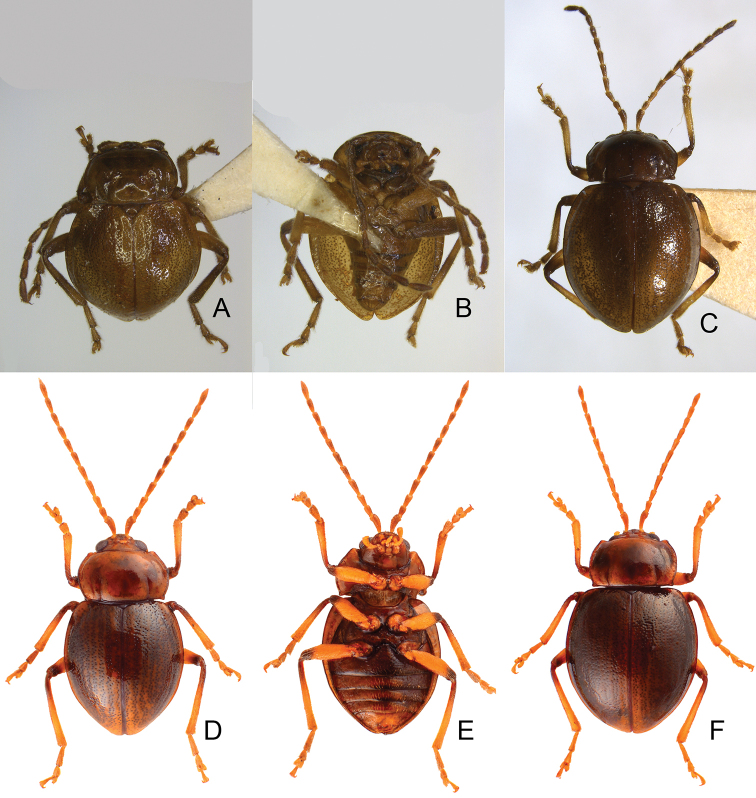
Habitus of *Taiwanoshaira chujoi* comb. nov. **A** male, paratype, from Alishan (阿里山), dorsal view **B** same, ventral view **C** female, paratype, from Tapan (達邦), dorsal view **D** male, from Bilu Divine Tree (碧綠神木), dorsal view **E** same, ventral view **F** female, from Bilu Divine Tree (碧綠神木), dorsal view.

Elytra ca 1.10–1.26 times as long as wide, almost glabrous, lateral margins rounded, apically tapering in males of *T. chujoi* comb. nov. and *T. tsoui* sp. nov., or both sexes of *T. taipingshanensis* sp. nov. Humeral calli reduced. Epipleura broad at base (Fig. 2D), gradually narrowed from base to basal 1/3, strongly narrowed at basal 1/3, abbreviated at apical 1/3. Disc with dense, confused punctures in *T. chujoi* comb. nov. (Fig. 3A, C–D, F) and *T. tsoui* sp. nov. (Fig. 6D, F), or with longitudinal impunctate ridges in *T. taipingshanensis* sp. nov. (Fig. 6A, C). Wingless, hindwings absent.

Ventral surface glabrous except abdomen, which is covered with pale setae. Anterior coxal cavities widely open (Fig. 2B) in *T. chujoi* comb. nov. and *T. tsoui* sp. nov., or almost closed (Fig. 2A) in *T. taipingshanensis* sp. nov. Prosternal process wide between procoxae. Abdomen simple, posterior margin of last ventrite (V) with median lobe in males (Fig. 2C); base extending anteriorly and almost reaching base of ventrite IV, median ridges present from base to apex of ventrite IV.

Legs slender. Tibiae lacking apical spines. Protarsomeres I slightly swollen in males of *T. taipingshanensis* sp. nov., but unmodified in either sex of *T. chujoi* sp. nov. and *T. tsoui* sp. nov. Metatarsomeres I a little longer than pro- and mesotarsomeres I, subequal to II and III combined. Claws appendiculate.

**Figure 4.**
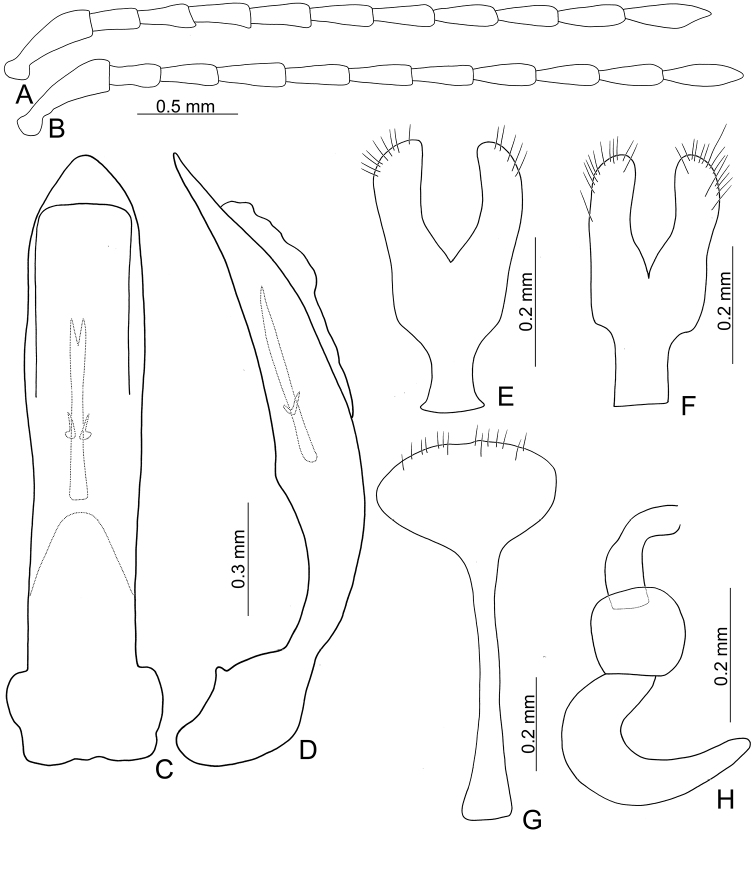
Diagnostic characters of *Taiwanoshaira chujoi* comb. nov. **A** antenna, male **B** antenna, female **C** penis, dorsal view **D** penis, lateral view **E** gonocoxae, from Tapan (達邦) **F** gonocoxae, from Pilu (碧綠) **G** abdominal ventrite VIII **H** spermatheca.

Penis (Figs 4C–D, 7C–G, 8C–L) slender, apex rounded; tectum broad, apical margin truncate; strongly curved in lateral view; internal sac with one longitudinal sclerite and one pair of small sclerites at sides near base of longitudinal sclerite.

Gonocoxae (Figs 4E–F, 7H–I, 8M–N) wide, tightly conjunct from base to middle; each gonocoxa wide, with four to 13 setae from near apex to apical 1/6, apex truncate or widely rounded; base irregular in *T. taipingshanensis* sp. nov. or narrowed in basal 1/3 in *T. chujoi* comb. nov. and *T. tsoui* sp. nov. Ventrite VIII (Figs 4G, 7J–K, 8O–P) well sclerotized, with several short setae at apex, spiculum elongate. Spermathecal receptaculum (Figs 4H, 7L, 8Q) strongly swollen; pump slender and curved; sclerotized spermathecal duct short.

###### Diagnosis.

Adults of *Taiwanoshaira* gen. nov. lack metathoracic wings, but elytra present, which completely cover the abdomen. Furthermore, the morphology of the elytra differs from that of *Shaira*. In *Shaira* the elytra possess an elongate ridge from the humeral area to the apex, dividing the elytra into a horizontal part and a lateral inclined part. The narrow epipleura are situated below this vertically inclined part of the elytra. In *Taiwanoshaira* gen. nov. the elytra possess a margin that separates the horizontal elytral surface from the epipleura, which are inclined.

###### Remarks.

The genus *Shaira* was proposed by Maulik (1936) for brachelytrous Galerucinae with appendiculate claws and slender antennae. Beenen (2013) argued that *Shaira* should be classified in Luperini. Kimoto (1982) described *S. chujoi* from Taiwan and compared it with *S. maculata* Maulik, 1936 from Manipur (India). *Shaira chujoi* somewhat resembles this species, but differs in having the body shorter and more oval, pronotum transverse and with different coloration. In fact, *S. chujoi* should not have been included in the *Shaira*, because it differs in possessing entire elytra but the ridge is absent. We transfer it to the new genus *Taiwanoshaira*.

*Taiwanoshaira* gen. nov. is likely not closely related to *Shaira*, although also included in Luperini. It is unique and, although apterous, likely to be more closely related to the genus *Sikkimia* Duvivier, 1891 with all bordered pronotal margins, two pairs of longitudinal furrows starting from base, one pair deeper and shorter near middle, the other pair longer but shallow near sides. This new genus is easily separated from *Sikkimia* with the open procoxal cavities and uniform antennae in both sexes (the closed procoxal cavities and modified antennomeres X and XI in males of *Sikkimia*).

###### Etymology.

The new genus name combines “Taiwan” and “*Shaira*” to indicate that this is a new genus endemic to Taiwan that is similar to *Shaira*. The gender is feminine.

###### Included species.

*Taiwanoshaira chujoi* (Kimoto), comb. nov., *T. taipingshanensis* sp. nov., and *T. tsoui* sp. nov.

##### Taiwanoshaira chujoi

Taxon classificationAnimaliaColeopteraChrysomelidae

(Kimoto) comb. nov.

2D8CDE21-0B08-527D-8685-AD3ACAAF91A3

Figures 3 , 4

Shaira chujoi Kimoto, 1982

###### Types.

***Holotype*** ♂ (KUEC): “Tapan [達邦] / Taiwan / 16. V. 1974 / S. TAKEDA [p, y] // Shaira / chujoi / Kimoto, n. sp. [h, w] // HOLOTYPE [p, r] // PHOTO [p, r] // 九大 [h, w, abbreviation for Kyushu University]”. ***Paratypes***: 1♂ (KMNH) (Fig. 3A, B): “(Taiwan) / Alishan (阿里山), 2300m / Chiayi Hsien [p, w] // 6.VI.1965 / T. Nakane [p, w] // Shaira / chujoi / Kimoto, n. sp. [h, w] // PARATYPE [p, b] // Japan-U. S. / Co-op. Sci. / Programme [p, y]”; 1♀ (KMNH) (Fig. 3C): “TAPAN [達邦] / TAIWAN / 16. V. 1974 / S. TAKEDA [p, y] // Shaira / chujoi / Kimoto, n. sp. [h, w] // PARATOPOTYPE [p, b]”.

###### Other material

**(*N* = 68). Hualien**: 1♀ (TARI), Bilu Divine Tree (碧綠神木), 10–11.X.2013, leg. K. Takahashi; 5♂♂, 4♀♀ (NMNS), same locality, 1.VI.-28.VII.2011; 2♂♂ (TARI), same locality (= Pilu), 13.VI.2014, leg. C.-F. Lee; 1♀ (TARI), same but with “leg T.-H. Lee”; 6♂♂, 12♀♀ (TARI), same locality, 30.VII.2014, leg. C.-F. Lee; 1♂ (TARI), same but with “leg. T.-H. Lee”; 8♂♂, 9♀♀ (TARI), same locality, 7.VII.2015, leg. C.-F. Lee; 4♂♂, 3♀♀ (TARI), Chian (吉安), 18.VI.2015, leg. T.-H. Lee; 1♂ (TARI), Kalapao (卡拉寶), 15–17.VII.2019, leg. B.-H. Ho; 4♂♂, 3♀♀ (3♂♂, 1♀TARI; 1♂, 2♀♀: RBCN), Kuanyuan (關原), 25.VIII.2014, leg. F.-S. Huang; 2♂♂, 1♀ (1♂, 1♀: TARI; 1♂: RBCN), Pilu (畢祿), 8.VIII.2014, leg. M.-H. Tsou; **Nantou**: 1♂ (NMNS), Meifeng (梅峰), 8.VII.-5.VIII.2003, leg. C. S. Lin & W. T. Yang; 1♂ (NMNS), Yuanfeng (鳶峰), 13.VIII.-10.IX.2002, leg. C. S. Lin & W. T. Yang; 1♂ **Taitung**: 1♂ (TARI), Liyuan (栗園), 5.X.2010, leg. T.-H. Lee.

###### Redescription.

Length 4.1–5.0 mm, width 2.5–3.3 mm. General color dark brown or blackish-brown (Fig. 3); each antennomere paler at base; legs yellowish-brown, but apices of femora and bases of tibiae blackish-brown. Antennae (Fig. 4A) filiform in males, ratios of lengths of antennomeres I to XI 1.0 : 0.5 : 0.6 : 0.6 : 0.6 : 0.6 : 0.6 : 0.6 : 0.6 : 0.7 : 0.9; ratios of length to width from antennomeres I to XI 3.3 : 2.3 : 2.5 : 2.7 : 2.7 : 2.9 : 2.9 : 3.0 : 2.9 : 3.1 : 3.6; similar in females, ratios of lengths of antennomeres I to XI (Fig. 4B) 1.0 : 0.5 : 0.6 : 0.7 : 0.6 : 0.6 : 0.6 : 0.6 : 0.6 : 0.6 : 0.8; ratios of length to width from antennomeres I to XI 3.2 : 2.2 : 2.7 : 2.9 : 3.2 : 3.1 : 3.0 : 2.8 : 3.0 : 3.0 : 3.6. Procoxal cavities widely open. Elytra 1.10–1.20 times longer than wide; disc with dense, confused, coarse punctures and longitudinal smooth patches; apices tapering in males but widely rounded in females. Tarsomeres I of front legs not modified in either sex. Penis (Fig. 4C, D) wide, about 6.3 times longer than wide; parallel sided but slightly curved in lateral view, apex narrowly rounded, base slightly sinuate; tectum broad from apical 1/10 to middle, apex truncate; ventral surface with large opening. Endophallic spiculae complex with median endophallic spiculae slender, apically bifurcate, and straight in lateral view, with one pair of small sclerites near basal third. Gonocoxae (Fig. 4E, F) short; apex of each gonocoxa widely rounded, with five to 13 long setae along apical margin, strongly narrowed in basal 1/3, with extreme wide base. Ventrite VIII (Fig. 4G) short and well sclerotized, with several short setae along apical margin, spiculum short. Spermathecal receptaculum (Fig. 4H) slightly swollen; pump extremely slender and curved; sclerotized spermathecal duct short.

###### Diagnosis.

Adults of *Taiwanoshaira chujoi* (Kimoto) comb. nov. are similar to those of *T. tsoui* sp. nov. based on the following shared characters: elytra smooth and lacking longitudinal ridges (Figs 3A, C–D, 6D, F) (presence of the longitudinal ridges on elytra in *T. taipingshanensis* sp. nov. (Fig. 6A, C)), widely open procoxal cavities (Fig. 2B) (almost closed procoxal cavities (Fig. 2A) in *T. taipingshanensis* sp. nov.), yellowish-brown legs with dark apices of femora and bases of tibiae (Figs 3, 6D–F) (entirely black legs in *T. taipingshanensis* sp. nov. (Fig. 6A–C)), uniform protarsi I in both sexes (sexually dimorphic protarsi I in *T. taipingshanensis* sp. nov.), tapering elytral apices in only males (Fig. 3) (tapering elytral apices of both sexes (Fig. 6A–C) in *T. taipingshanensis* sp. nov.). *Taiwanoshaira chujoi* comb. nov. differs from *T. tsoui* sp. nov. in possessing black or blackish elytra (Fig. 3) with denser punctures in contrast to the black or blackish-brown elytra with yellowish-brown suture and margin (Fig. 6D–F) and sparser punctures of *T. tsoui* sp. nov. In addition, most genitalic characters of this species are diagnostic, including the slightly curved penis (Fig. 4C, D) (moderately curved (Fig. 8C, D) in *T. tsoui* sp. nov.), wider base of gonocoxae (Fig. 4E–F) (narrower base of gonocoxae (Fig. 8M, N) in *T. tsoui* sp. nov.), and longer, slender spermathecal pump (Fig. 4H) (shorter, wider spermathecal pump (Fig. 8Q) in *T. tsoui* sp. nov.).

###### Host plants.

Probably some species of moss, currently unknown (Fig. 1B).

###### Distribution.

**Figure 5.**
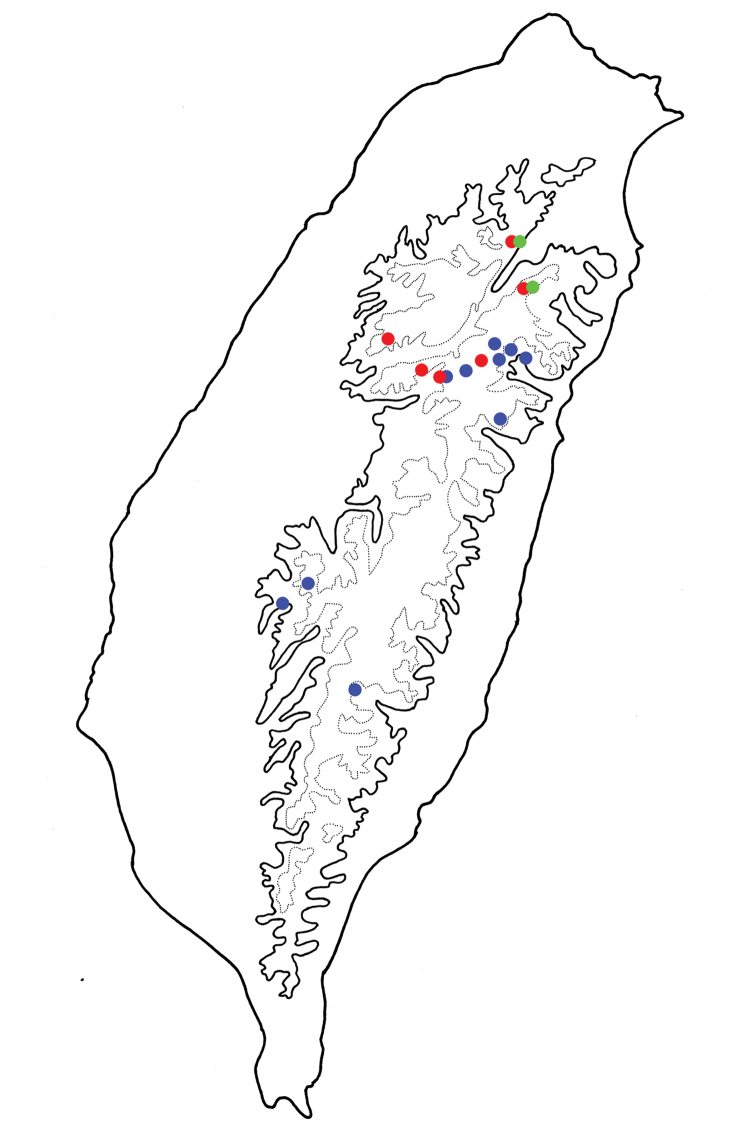
Distribution map of *Taiwanoshaira* species, solid line: 1000 m, broken line: 2000 m. **Blue Dots***T. chujoi* comb. nov. **Red Dots***T. tsoui* sp. nov. **Green Dots***T. taipingshanensis* sp. nov.

South and central Taiwan (Fig. 5). This species is sympatric with *T. tsoui* sp. nov. at Meifeng (梅峰).

##### Taiwanoshaira taipingshanensis sp. nov.

Taxon classificationAnimaliaColeopteraChrysomelidae

F7427B54-1E09-54FA-802D-255150E29E03

http://zoobank.org/1AE3EA77-43D4-420C-A3F3-E92F3CA65A9B

Figures 6A–C , 7

###### Types

**(*N* = 77). *Holotype*** ♂ (TARI): Taiwan. Ilan: Taipingshan (太平山), 5.VIII.2015, leg. Y.-T. Chung. ***Paratypes*.** 5♂♂, 15♀♀ (TARI), same as holotype; 10♂♂, 16♀♀ (TARI), Yuanyanghu (鴛鴦湖), 19.VIII.2010, leg. S.-S. Li; 4♂♂, 5♀♀ (TARI), same locality, 22.VIII.2011, leg. C.-F. Lee; 2♂♂, 8♀♀ (2♀♀: TARI; 2♂♂, 2♀♀: RBCN), same but with “leg. M.-H. Tsou”; 5♂♂, 6♀♀ (TARI), same but with “leg. H. Lee”.

###### Description.

**Figure 6.**
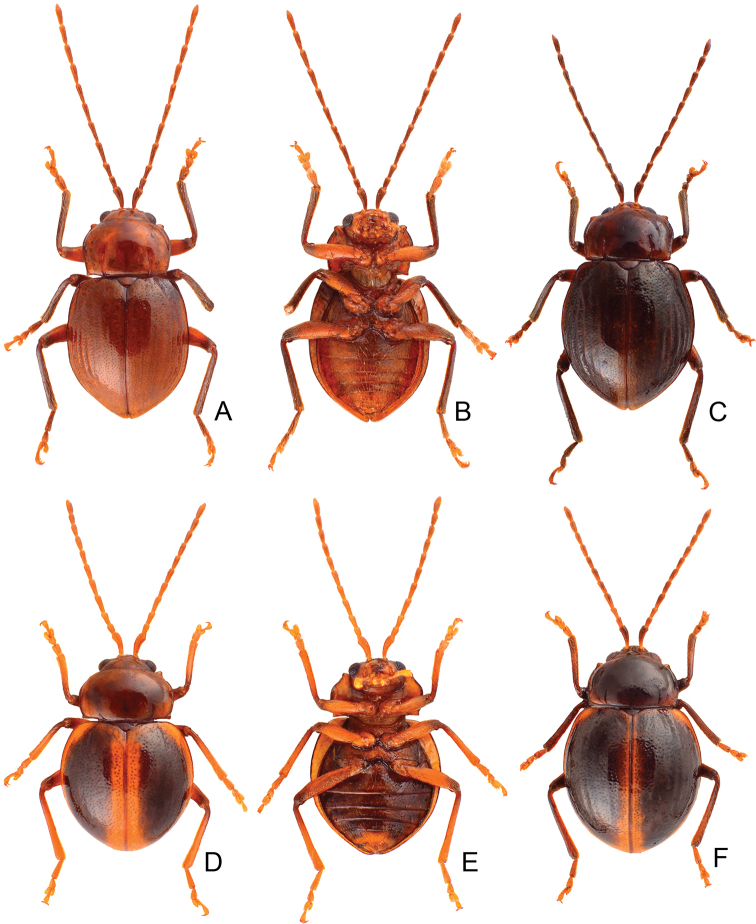
Habitus of *Taiwanoshaira taipingshanensis* sp. nov. and *T. tsoui* sp. nov. **A***T. taipingshanensis* sp. nov., male, dorsal view **B** same, ventral view **C** same, female, dorsal view **D***T. tsoui* sp. nov., male, dorsal view **E** same, ventral view **F** same, female, dorsal view.

**Figure 7.**
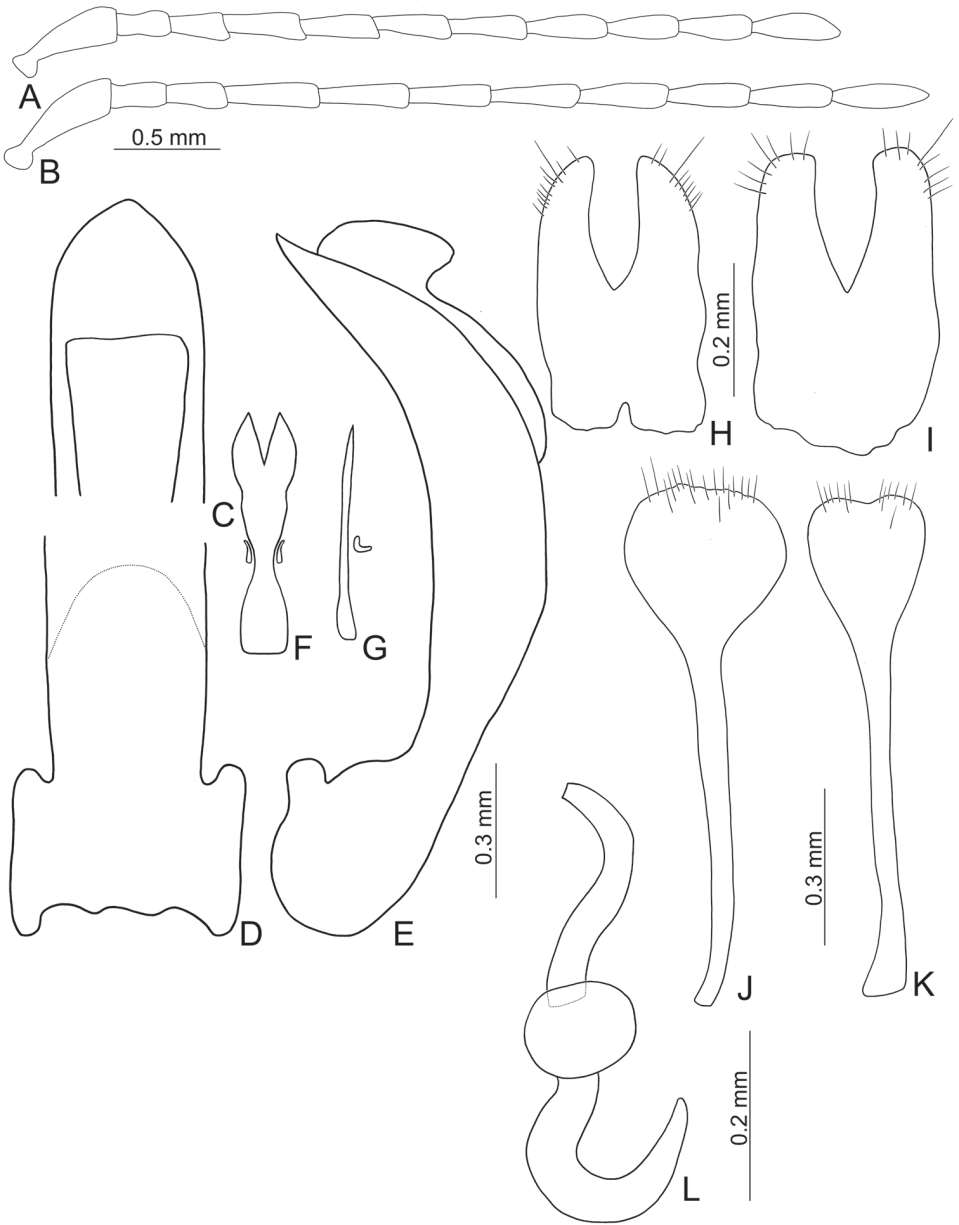
Diagnostic characters of *Taiwanoshaira taipingshanensis* sp. nov. **A** antenna, male **B** antenna, female **C** penis, apex, dorsal view **D** penis, base, dorsal view **E** penis, lateral view **F** endophallic sclerites, dorsal view **G** ditto, lateral view **H** gonocoxae, from Yuanyanghu (鴛鴦湖) **I** same, from Taipingshan (太平山) **J** abdominal ventrite VIII, from Yuanyanghu (鴛鴦湖) **K** same, from Taipingshan (太平山) **L** spermatheca.

Length 4.0–5.7 mm, width 2.6–3.4 mm. General color dark brown or blackish-brown (Fig. 6A–C). Antenna (Fig. 7A) filiform in males, ratios of lengths of antennomeres I to XI 1.0 : 0.5 : 0.6 : 0.7 : 0.7 : 0.7 : 0.7 : 0.7 : 0.6 : 0.7 : 0.8; ratios of lengths to widths from antennomeres I to XI 3.0 : 2.0 : 2.2 : 3.0 : 3.2 : 3.2 : 3.3 : 3.5 : 3.0 : 3.1 : 3.4; similar in females, ratio of lengths of antennomeres I to XI (Fig. 7B) 1.0 : 0.4 : 0.5 : 0.8 : 0.7 : 0.7 : 0.7 : 0.7 : 0.7 : 0.7 : 0.8; ratios of lengths to widths from antennomeres I to XI 3.4 : 2.3 : 2.3 : 3.9 : 3.7 : 3.7 : 4.0 : 3.7 : 3.4 : 3.5 : 4.2. Pronotum 1.61–1.63 times wider than long; lateral margins slightly rounded; disc with fine punctures bearing tiny setae. Procoxal cavities almost closed. Elytra 1.11–1.20 times longer than wide; disc with dense, coarse punctures and longitudinal ridges not covered with punctures; apices tapering in both sexes. Tarsomeres I of front legs slightly swollen in males, not modified in females. Penis (Fig. 7C–E) wide, about 5.5 times longer than wide; parallel sided and strongly curved in lateral view, apex narrowly rounded, base with shallow median notch; tectum broad from apical 1/6 to middle, apex truncate; ventral surface with large opening. Endophallic spiculae complex (Fig. 7F, G) with median endophallic spiculae slender, apically bifurcate, and straight in lateral view; with one pair of small sclerites near base. Gonocoxae (Fig. 7H, I) short; apex of each gonocoxa widely rounded, with eight to ten long setae along apical margin, basal margin irregular. Ventrite VIII (Fig. 7J, K) short and well sclerotized, with several short setae along apical margin, spiculum long. Spermathecal receptaculum (Fig. 7L) strongly swollen; pump extremely slender and curved; sclerotized spermathecal duct long.

###### Variation.

Female genitalic characters are variable among different localities. The apices of the gonocoxae are widely rounded in specimens from Taipingshan (太平山) (Fig. 7I) but tapering in those from Yuanyanhu (鴛鴦湖) (Fig. 7H). The apex of abdominal ventrite VIII is shorter in specimens from Taipingshan (Fig. 7K) than those from Yuanyanhu (Fig. 7J).

###### Diagnosis.

**Figure 8.**
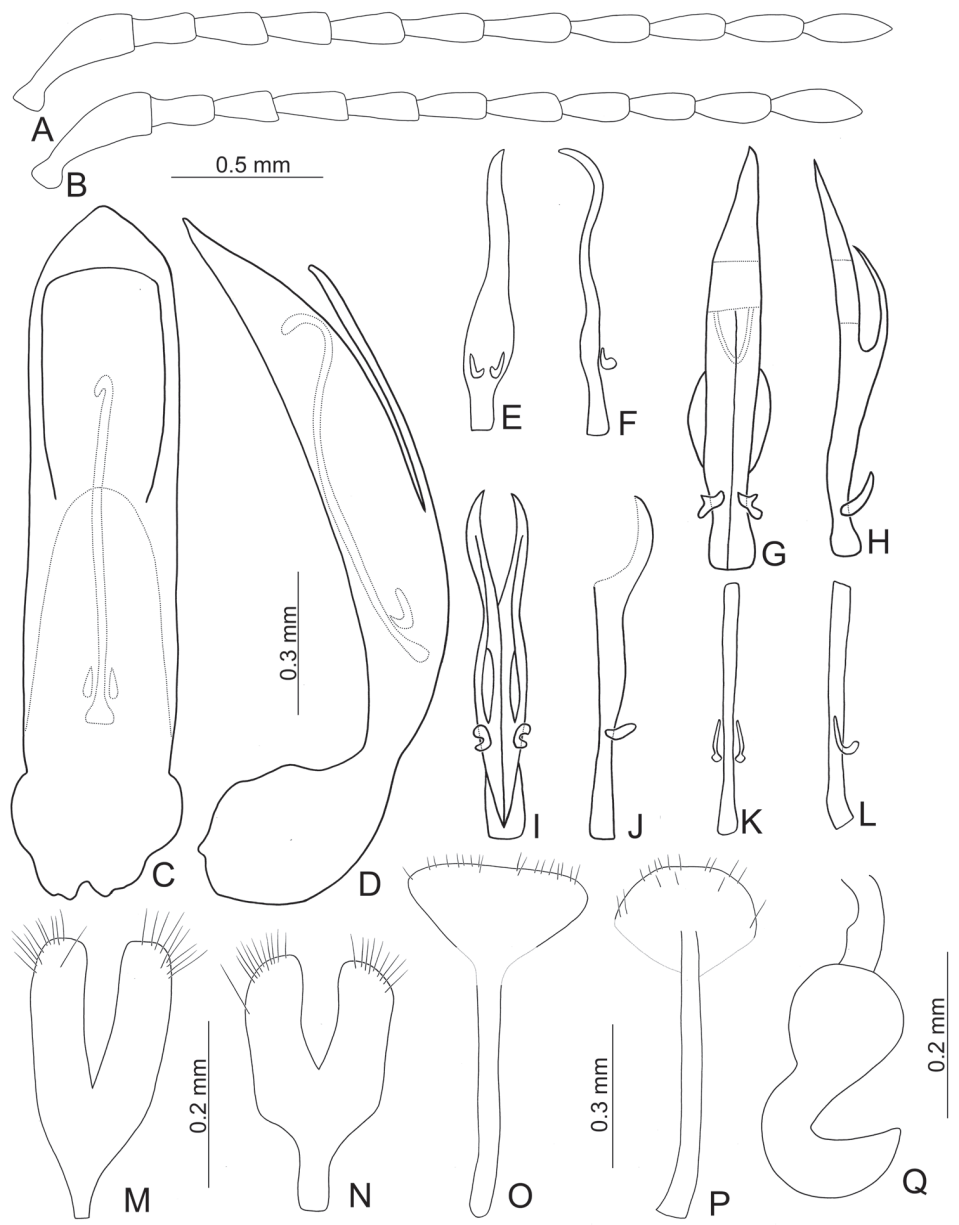
Diagnostic characters of *Taiwanoshaira tsoui* sp. nov. **A** antenna, male **B** antenna, female **C** penis, dorsal view **D** penis, lateral view **E** endophallic sclerites, from Meifeng (梅峰), dorsal view **F** ditto, lateral view **G** same, from Peitungyanshan (北東眼山), dorsal **H** ditto, lateral view **I** samel, from Tahsuehshan (大雪山), **J** ditto, laeral view **K** same, form Taipingshan (太平山), dorsal **L** ditto, lateral view **M** gonocoxae, from Hsiaofengkou (小風口) **N** same, from Taipingshan (太平山) **O** abdominal ventrite VIII, from Peitungyanshan (北東眼山) **P** same, from Taipingshan (太平山) **Q** spermatheca.

*Taiwanoshaira taipingshanensis* sp. nov. is easily separated from other congeners by the presence of longitudinal ridges on the elytra (Fig. 6A, C) (lacking longitudinal ridges in others (Figs 3A, C–D, F; 6D, F)), almost closed procoxal cavities (Fig. 2A) (widely open procoxal cavities (Fig. 2B) in others), entirely black legs (Fig. 6A–C) (yellowish-brown legs with dark apices of femora and bases of tibiae in others (Figs 3, 6D–F)), sexually dimorphic protarsi I (uniform protarsi I in both sexes of others), tapering elytral apices of both sexes (Fig. 6A–C) (tapering elytra apices in only males of others (Figs 3, 6D–F)). In addition, most genitalic characters of this new species are diagnostic, including the extremely strongly curved penis (Fig. 7C–E) (moderately or slightly curved in others (Figs 4C, D; 8C, D), irregular base of gonocoxae (Fig. 7H, I) (narrowed base of gonocoxae in others (Figs 4E, F; 8M, N)), and long sclerotized spermathecal duct (Fig. 7L) (short sclerotized spermathecal duct in others (Figs 4H, 8Q)).

###### Host plants.

Mniaceae: *Plagiomnium vesicatum* (Besch.) T.J. Kop. We observed that adults fed on leaves of host plants (Fig. 1C, D).

###### Etymology.

This new species is named for its type locality.

###### Distribution.

Known from two localities in northern Taiwan (Fig. 5). This new species is sympatric with *T. tsoui* sp. nov.

##### Taiwanoshaira tsoui sp. nov.

Taxon classificationAnimaliaColeopteraChrysomelidae

9E8866E4-A7FD-5B04-87C2-231B2E656289

http://zoobank.org/8B744065-FF3A-41D4-ADD4-9952A41D0D7F

Figures 6D–F , 8

###### Types

**(*N* = 54). *Holotype*** ♂ (TARI): Taiwan. Nantou: Hsiaofengkou (小風口), 9.VIII.2012, leg. C.-F. Lee. ***Paratypes.*** 14♂♂, 21♀♀ (12♂♂, 19♀♀TARI; 2♂♂, 2♀♀: RBCN), same data as holotype; 7♂♂, 6♀♀ (TARI), same but with “leg. T.-H. Lee”; 2♂♂, 2♀♀ (TARI), same locality, 29.VII.2014, leg. C.-F. Lee; 1♀ (NMNS), same locality, 23.VI.–24.VIII.2009, leg. W. T. Yang & K. W. Huang; 1♂, 3♀♀ (NMNS), same locality, 24.VIII.–24.IX.2009, leg. W. T. Yang & K. W. Huang; Ilan: 2♂♂, 1♀ (TARI), Taipingshan (太平山), 5.VIII.2015, leg. Y.-T. Chung; 1♀ (TARI), Yuanyanghu (鴛鴦湖), 19.VIII.2010, leg. S.-S. Li; Nantou: 1♀ (TARI), Meifeng (梅峰), 11.VI.2014, leg. C.-F. Lee; 1♂ (TARI), same locality, 29.VII.2014, leg. C.-F. Lee; 9♂♂, 2♀♀ (TARI), Peitungyanshan (北東眼山), 3.VII.2014, leg. C.-F. Lee; Taichung: 8♂♂, 4♀♀ (TARI), Tahsuehshan (大雪山), 2.VIII.2019, leg. B.-X. Guo.

###### Description.

Length 4.1–4.8 mm, width 2.5–2.9 mm. General color dark brown or blackish-brown (Fig. 6D–F); each antennomere basally paler; margins of pronotum and elytra, including suture yellowish-brown; legs yellowish-brown but apices of femora and bases of tibiae dark brown. Antennae (Fig. 8A) filiform in males, ratio of lengths of antennomeres I to XI 1.0 : 0.5 : 0.5 : 0.5 : 0.5 : 0.6 : 0.7 : 0.6 : 0.6 : 0.6 : 0.7; ratios of lengths to widths from antennomeres I to XI 3.3 : 2.2 : 2.3 : 2.2 : 2.5 : 2.7 : 3.1 : 2.8 : 2.8 : 2.7 : 3.2; similar in females, ratio of lengths of antennomeres I to XI (Fig. 8B) 1.0 : 0.5 : 0.5 : 0.5 : 0.5 : 0.5 : 0.5 : 0.5 : 0.5 : 0.5 : 0.7; ratios of lengths to widths from antennomeres I to XI 3.2 : 2.1 : 2.0 : 2.4 : 2.5 : 2.7 : 2.6 : 2.4 : 2.4 : 2.4 : 2.7. Pronotum 1.63–1.68 times wider than long; lateral margins moderately rounded; disc with fine punctures bearing tiny setae. Procoxal cavities widely open. Elytra 1.17–1.26 times longer than wide; disc with sparse, confused, fine punctures; apices tapering in males, but widely rounded in females. Protarsomeres I not sexually dimorphic. Penis (Fig. 8C, D) wide, about 5.6 times longer than wide; parallel sided and moderately curved in lateral view, apex narrowly rounded, base with shallow median notch; tectum broad from apical 1/6 to middle, apex truncate; ventral surface with large opening. Endophallic spiculae complex with median endophallic spiculae extremely slender, apically curved in lateral view; with one pair of small sclerites near base. Gonocoxae (Fig. 8M, N) short; apex of each gonocoxa widely rounded, with eight to 11 long setae along apical margin, basally narrowed. Ventrite VIII (Fig. 8O, P) short and well sclerotized, with several short setae along apical margin, spiculum short. Spermathecal receptaculum (Fig. 8Q) swollen; pump slender and curved; sclerotized spermathecal duct short.

###### Variation.

Specimens from Hsiaofengkou (小風口) have paler bodies and shorter antenna than others. The endophallic spiculae complexes are variable among localities: subbases of endophallic spiculae are shorter and wider in specimens from Meifeng (梅峰) (Fig. 8E, F); similar to those from Meifeng but with a median membranous area and straight apex in specimens from Peitungyanshan (北東眼山) (Fig. 8G, H); similar to those in Peitungyangshan, but with bifurcate apices in specimens from Tahsuehshan (大雪山) (Fig. 8I, J); specimens from Taipingshan (太平山) (Fig. 8K, L) possess more slender median endophallic spiculae than those from Hsiaofengkou and shorter more truncate apices. Females from Hsiaofengkou have gradually narrowed bases of the gonocoxae (Fig. 8M) that differ from those with strongly narrowed bases in others (Fig. 8N). Females from Taipingshan have narrower apices of abdominal ventrites VIII (Fig. 8P) than others (Fig. 8O). It raises the question whether such variations of endophallic spiculae complexes at different localities indicate interspecific differentiation since endophallic sclerites are usually very consistent within a species. The problem needs further study by collecting more material from additional localities and combined with molecular study.

###### Diagnosis.

Adults of *T. tsoui* sp. nov. are similar to those of *T. chujoi* (Kimoto) comb. nov. in sharing the following characters: elytra smooth and lacking longitudinal ridges (Figs 3A, C, D, F; 6D, F) (presence of the longitudinal ridges on elytra (Fig. 6A, C) in *T. taipingshanensis* sp. nov.), widely open procoxal cavities (Fig. 2B) (almost closed procoxal cavities (Fig. 2A) in *T. taipingshanensis* sp. nov.), yellowish-brown legs with dark apices of femora and bases of tibiae (Figs 3, 6D–F) (entirely black legs (Fig. 6A–C) in *T. taipingshanensis* sp. nov.), uniform protarsi I in both sexes (sexually dimorphic protarsi I in *T. taipingshanensis* sp. nov.), tapering elytra apices only in males (Figs 3, 6D–F) (tapering elytral apices of both sexes (Fig. 6A–C) in *T. taipingshanensis* sp. nov.). Adults of *T. tsoui* sp. nov. differ from those of *T. chujoi* comb. nov. by possessing yellowish-brown sutures and margins with black or blackish-brown elytra having punctures more sparse (Fig. 6D, F), in contrast to black or blackish elytra (Fig. 3A, C) with denser punctures in *T. chujoi* comb. nov. In addition, most genitalic characters of this species are diagnostic, including moderately curved penis (Fig. 8C, D) (slightly curved (Fig. 4C, D) in *T. chujoi* comb. nov.), narrower base of gonocoxae (Fig. 8M, N) (wider base of gonocoxae (Fig. 4E, F) in *T. chujoi* comb. nov.), and shorter spermathecal pump (Fig. 8Q) (much longer pump (Fig. 4H) in *T. chujoi* comb. nov.).

###### Food plants.

Probably some species of moss, currently unknown (Fig. 1E, F).

###### Etymology.

This new species is dedicated to Mei-Hua Tsou, a member of TCRT and the first to collect this new species.

###### Distribution.

Northern and central Taiwan (Fig. 5). It is sympatric with *T. taipingshanensis* sp. nov. at Yuanyanahu (鴛鴦湖) and Taipingshan (太平山), and with *T. chujoi* comb. nov. at Meifeng (梅峰).

### Key to species of the new genus *Taiwanoshaira*

**Table:** 

1	Elytra with longitudinal ridges, apically narrowed in both sexes (Fig. 6A, C); procoxal cavities almost closed (Fig. 2A); legs entirely black or blackish (Fig. 6A–C), protarsi I swollen in males	***T. taipingshanensis* sp. nov.**
–	Elytra smooth, without longitudinal ridges, apically narrowed in males but widely rounded in females (Figs 3, 6D, F); procoxal cavities widely open (Fig. 2B); legs yellowish-brown but apices of femora and bases of tibiae darker (Figs 3, 6D–F), protarsi I not modified in either sex	**2**
2	Elytra entirely black (Fig. 3A, C, D, F), punctures on disc denser	***T. chujoi* (Kimoto) comb. nov.**
–	Elytra with yellowish-brown sides and suture (Fig. 6D, F), punctures on disc sparser	***T. tsoui* sp. nov.**

## Discussion

Mosses are common all over Taiwan since it is a country with high humidity. They are most dominant in cloud forests. The montane cloud forest of Taiwan was mapped using 12-year MODIS-derived ground fog frequency data (Schulz et al. 2017). It covers most montane areas above 1000 m altitude. However, species of *Taiwanoshaira* are restricted to limited areas based on TCRT’s collecting experience. Moreover, they were absent at some localities where they were recorded 40 years ago, such as Tapan (達邦) and Alishan (阿里山) for *T. chujoi* comb. nov. They are currently common at only a few places, including Yuanyang Lake (鴛鴦湖), Hsiaofengkou (小風口), and Bilu Divine Tree (碧綠神木). Of these localities, the climatic characters of the cloud forest at Yuanyang Lake (鴛鴦湖) was studied from 1994 to 2004 (Lai et al. 2006). This site (24°N, 121°24'E) is situated in Chi-Lan Mountain at an elevation of 1650 to 2420 m above sea level. The annual mean air temperature was 12.7 °C. The lowest mean monthly temperature was during February (monthly mean 5.9 °C), and the highest during July (month mean 18.1 °C). Winter featured light rain with a prolonged occurrence of fog, resulting in a large reduction of solar radiation. In summer, fog typically occurred during early morning and again from afternoon to evening. The latter was associated with wind direction changes and was usually accompanied by short moderate to heavy convective rain. The relative humidity was usually higher than 90%. The annual precipitation varied between 2109 mm (in 1995) and 4727 mm (in 2001), with an average of 3396 mm. On average, there were 239 rainy days per year. These climatic characteristics indicate that *Taiwanoshaira* species occur in microhabitats with high humidity year round. Adults of *Taiwanoshaira* were almost absent in south Taiwan except that one specimen was collected at Liyuan (栗園), Taitung county. This implies that the species might not survive in places where the climate has changed greatly, even though mosses persist and grow well.

## Supplementary Material

XML Treatment for Taiwanoshaira

XML Treatment for Taiwanoshaira chujoi

XML Treatment for Taiwanoshaira taipingshanensis

XML Treatment for Taiwanoshaira tsoui
